# RNA-seq Reveals the Overexpression of IGSF9 in Endometrial Cancer

**DOI:** 10.1155/2018/2439527

**Published:** 2018-02-14

**Authors:** Zonggao Shi, Chunyan Li, Laura Tarwater, Jun Li, Yang Li, William Kaliney, Darshan S. Chandrashekar, M. Sharon Stack

**Affiliations:** ^1^Harper Cancer Research Institute, University of Notre Dame, South Bend, IN 46617, USA; ^2^Department of Chemistry and Biochemistry, University of Notre Dame, South Bend, IN 46617, USA; ^3^Department of Gynecology and Obstetrics, Shandong Provincial Hospital Affiliated to Shandong University, Jinan, Shandong Province, China; ^4^Department of Applied and Computational Mathematics and Statistics, University of Notre Dame, South Bend, IN 46617, USA; ^5^Department of Pathology, The Medical Foundation, South Bend, IN 46601, USA; ^6^Department of Pathology, University of Alabama School of Medicine, Birmingham, AL 35294, USA

## Abstract

We performed RNA-seq on an Illumina platform for 7 patients with endometrioid endometrial carcinoma for which both tumor tissue and adjacent noncancer tissue were available. A total of 66 genes were differentially expressed with significance level at adjusted *p* value < 0.01. Using the gene functional classification tool in the NIH DAVID bioinformatics resource, 5 genes were found to be the only enriched group out of that list of genes. The gene* IGSF9 *was chosen for further characterization with immunohistochemical staining of a larger cohort of human endometrioid carcinoma tissues. The expression level of IGSF9 in cancer cells was significantly higher than that in control glandular cells in paired tissue samples from the same patients (*p* = 0.008) or in overall comparison between cancer and the control (*p* = 0.003). IGSF9 expression is higher in patients with myometrium invasion relative to those without invasion (*p* = 0.015). Reanalysis of RNA-seq dataset from The Cancer Genome Atlas shows higher expression of IGSF9 in endometrial cancer versus normal control and expression was associated with poor prognosis. These results suggest IGSF9 as a new biomarker in endometrial cancer and warrant further studies on its function, mechanism of action, and potential clinical utility.

## 1. Introduction

Precision medicine calls for better characterization of diseases at molecular level. As the cost of next-generation sequencing (NGS) technology becomes increasingly affordable, more and more efforts have been invested on genome wide observation of genetic changes and gene expression profiles in cancer. NGS-based RNA-sequencing (RNA-seq) is an established and effective screening method in identifying new biomarkers and better understanding cancer biology [1]. Nationwide and international consortia like The Cancer Genome Atlas (TCGA) and the International Cancer Genomics Consortium (ICGC) have generated tremendous amounts of new data and new insights regarding the molecular landscape of many major types of cancer using various high throughput profiling technologies including RNA-seq [2–4].

Endometrial cancer of the uterus is responsible for about 74,000 deaths of women each year worldwide [5, 6]. It is the most common gynecological malignancy in America and other western countries. Among the histologic subtypes, endometrioid carcinoma is the most prevalent, accounting for 75%–85% of all patients. Other types, like serous, mucinous, clear cell and squamous cell carcinomas, are much less common. According to the data from SEER (Surveillance, Epidemiology, and End Results) program at the National Cancer Institute (https://seer.cancer.gov/), although the current survival rate of women with endometrial cancer is as high as 81%, annual deaths from endometrial cancer still numbered over 10,000 patients in the United States alone. Furthermore, for over three decades, the age-adjusted death rate of endometrial cancer in the general population has not decreased. Thus, it is imperative to better characterize the molecular details of endometrial cancer using the latest high throughput profiling technology to identify new biomarkers that may enhance our understanding of disease progression and may impact clinical management of this cancer.

The largest effort in interrogating the transcriptome of endometrial cancer is from TCGA [7]; however both the initial comprehensive report from TCGA on endometrial cancer and the follow-up report by others who focused on the RNA-seq data of endometrial cancer in TCGA did not provide transcriptome-wide information on the differentially expressed genes in the scenario of endometrial cancer versus normal endometrial tissue [7, 8]. Another effort by Xiong et al. examined the transcriptome profiles of endometrial cancer versus adjacent noncancer tissue, but with 3 patients only [9]. In the current study, we screened paired human endometrioid carcinoma tissues and the adjacent nontumor tissues from 7 patients using RNA-seq technology and compared their gene expression profiles. Further validation was performed by quantitative PCR and immunohistochemistry. Focusing on the overexpressed gene* IGSF9*, we explored its associations with clinicopathologic characteristics in endometrioid endometrial carcinoma.

## 2. Materials and Methods

### 2.1. Human Tissue Specimens

Two different approaches were used in human tissue specimen procurement. For RNA-seq, freshly harvested, RNAlater-preserved cancer and adjacent noncancer tissues were obtained from 7 patients through frozen section rooms at The Medical Foundation (South Bend, IN). For the follow-up immunohistochemistry study, formalin-fixed and paraffin-embedded (FFPE) blocks from a total of 56 patients were obtained from The Medical Foundation surgical archive. All specimens were collected during the period from 2013 to 2015 via the Harper Cancer Research Institute Tissue Biorepository project. All collection of human tissues from surgically removed uterine endometrial cancer specimens were conducted by The Medical Foundation pathologists. Pathologic diagnoses and classification were initially made by the pathologist on-duty and further reviewed by board-certified pathologists. Informed consent was obtained from all patients. Protocols were approved by the University of Notre Dame Institutional Review Board (IRB) and local hospital IRBs.

### 2.2. RNA-Sequencing and Data Processing

For RNA preparation, ~30 mg tissue from each specimen was homogenized using an Omni-TH tissue homogenizer (OMNI International, Kennesaw, GA) in prechilled lysis buffer from the AllPrep RNA/DNA/protein mini kit (Qiagen, Germantown, MD) according to the manufacturer's instructions. RNA elution was then quantified with a Nanodrop spectrophotometer (Thermo Fisher) and further analyzed by BioAnalyzer (Agilent) for quality control. Samples with RIN > 7 were used in RNA-seq library preparation by the Notre Dame Genomics Core Facility. RNA-seq libraries were prepared with nonstranded TruSeq RNA kit from Illumina (San Diego, CA) and sequenced on a HiSeq 4000 sequencer (BGI Hong Kong). The reads generated were paired-end and of length 100-nt. Raw reads were cleaned up by BGI with SOAPnuke to remove adapters and low-quality reads. All other command line software tools were installed and used on the high performance computer cluster at University of Notre Dame Center for Research Computing. FastQC (version 0.11.2) was used to examine the read quality. No further read trimming or filtering was performed. Splicing aware mapping tool, STAR (version 2.4.2a) was used for alignment in 2-pass mode. The resulting BAM files were examined by Qualimap (version 2.2) and then used to count reads per gene with the htseq-count function from HTSeq software (version 0.6.1).

R/Bioconductor packages including DESeq2 were used for gene expression analysis [10–12]. Dealing with paired samples, we used a multifactor design which includes the sample information by putting the condition of interest (cancer or noncancer) at the end of the design. When the data was treated as from two independent groups, only single factor design was used. For comparison, we also downloaded and reanalyzed the raw RNA-seq data published by Xiong et al. [9] from SRA (NIH Sequence Read Archive) with our own data processing protocol as mentioned above. To identify the most overrepresented biological terms and genes, we made use of the gene classification tool from DAVID Bioinformatics Resources 6.8 [13] (https://david.ncifcrf.gov). High classification stringency was used.

To access tumor subgroup gene expression and survival data in The Cancer Genome Atlas (TCGA) project, we used the web interface of UALCAN [14] and part of the processed data from UALCAN. UALCAN uses TCGA level 3 RNA-seq and clinical data from 33 cancer types. The expression level was normalized as transcripts per million reads (TMP) for comparison across groups and individuals.

### 2.3. Quantitative PCR

Taqman® gene expression assays were used in quantitative PCR. The primers and probe set for the* IGSF9* gene and reference control gene* ACTB* were bought from Life Technologies (Carlsbad, CA). RNA sample preparation and cDNA synthesis used TRIzol reagent and SuperScript II reagent, respectively, from Life Technologies. The q-PCR reaction was run on an ABI StepOnePlus real time thermal cycler (Life Technologies). Quantification was calculated with the ΔΔCt method using control samples and the expression of the reference gene* ACTB *for normalization [15].

### 2.4. Immunohistochemistry

The immunohistochemistry procedure is as previously described [16]. FFPE tissue sections (5 *μ*m in thickness) were dewaxed in xylene and then rehydrated. Heat induced epitope retrieval was performed with citrate buffer (pH 6.0). Primary antibodies for IGSF9 (catalog number: NBP1-93676, 1 : 200 diluted) and Ki67 (catalog number: ab15580, 1 : 500 diluted) were bought from NOVUS Biologicals (Littleton, CO) and Abcam (Cambridge, MA), respectively. Polymer-based chromogen visualization system used the ImmPRESS™ HRP anti-rabbit IgG product from Vector Laboratories (Burlingame, CA).

### 2.5. Image Analysis and Quantification

All immunohistochemistry slides were scanned and analyzed with the digital pathology system, including Aperio ScanScope CS, eSlide manager, and image analysis tools, made by Leica Biosystem (Chicago, Illinois). Quantification of IGSF9 used a macro built from the pixel counting algorithm (version 1), which reports the percentage of total number of positively stained pixels over total number of pixels analyzed. Cellular quantification nuclear stain algorithm (version 9) was used to make a macro to count positive cell percentage of total number of tumor cells analyzed in Ki67 immunostains.

### 2.6. Statistical Analysis

Rstudio and related R packages were used to perform all the statistical analyses involving RNA-seq data downstream processing, clinical pathological data, and IHC results. Significance level was set at *p* value < 0.05, while in screening the long gene lists from RNA-seq, adjusted *p* values were described in the results. Principle component analysis was used in analyzing the variance in overall RNA-seq counts. Wald test was used when comparing differential gene expression as implemented in DESeq2 package. When comparing means of two groups in clinical and IHC, nonparametric method Wilcoxon rank sum test (also known as Mann–Whitney *U* test) was used. Correlation tests used Pearson's method as implemented in base R and the* psych* package. Survival analysis used Kaplan-Meier method and log-rank test.

## 3. Results

### 3.1. Gene Expression Profiles of Endometrioid Endometrial Carcinoma and Benign Adjacent Tissues via RNA-seq

RNA-seq was performed for a total of 7 patients with both cancer tissues and adjacent benign endometrial tissues available. Patient ages ranged from 48 to 78. All specimens were from the same major histological type, endometrioid endometrial carcinoma; two patients were at histological grade I and five at grade II, all at clinical stage I (FIGO, 2009). On average, 17.2 million reads were produced from each of the 14 libraries. Sequencing read quality was examined by FastQC, all with a Phred score above 20. Alignment or mapping quality metrics as produced by Qualimap over the output BAM files from alignment with STAR are listed in Suppl.  Table 1.

**Figure 1:**
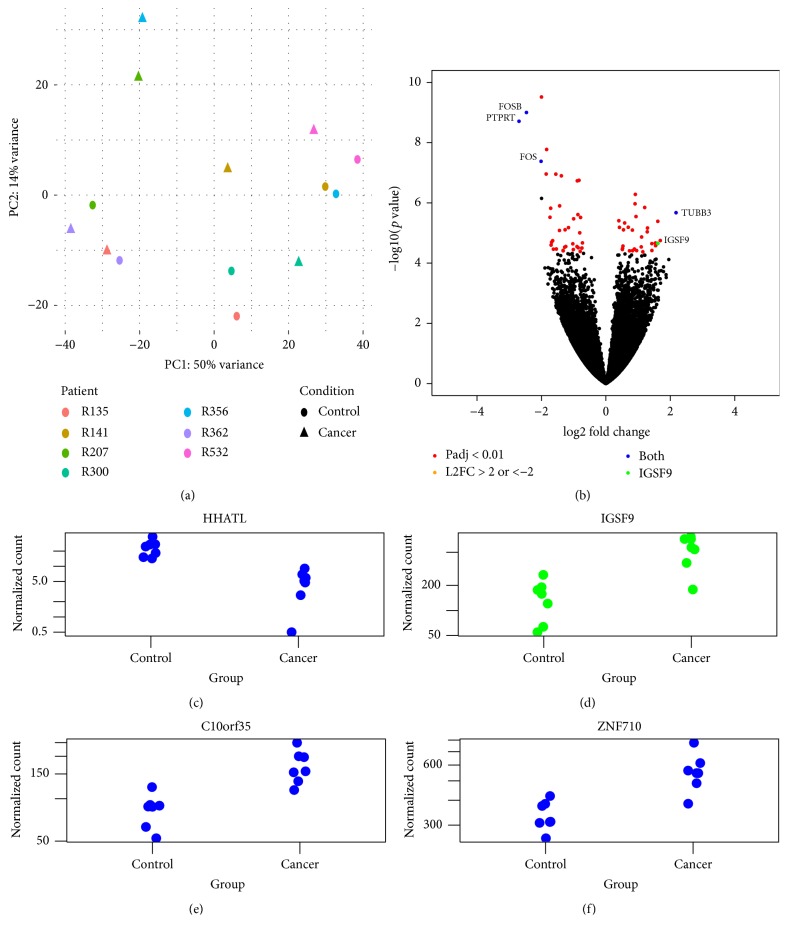
*RNA-seq identifies differentially expressed genes in endometrioid carcinoma*. (a) Principle component analysis showing the distance of variance among all the 14 samples from 7 patients, no obvious clustering for cancer versus noncancerous control; (b) volcano plot depicting the number of differentially expressed genes based on their *p* values and log2 fold change in the analysis of 7 pairs of samples; (c), (d), (e), and (f) normalized counts from the top 4 genes obtained by comparing the control and cancer tissues as two independent groups.

While exploring the gene count data, plotting the top two components from principle component analysis did not reveal any clustering trend in the 7 cancer samples versus the 7 controls (Figure 1(a)). Using DESeq2 as the main tool, we sought to find the differential expression of genes in cancer versus the control with two different approaches: (1) using the pairing information in the samples, (2) without considering the pairing information.

**Figure 2:**
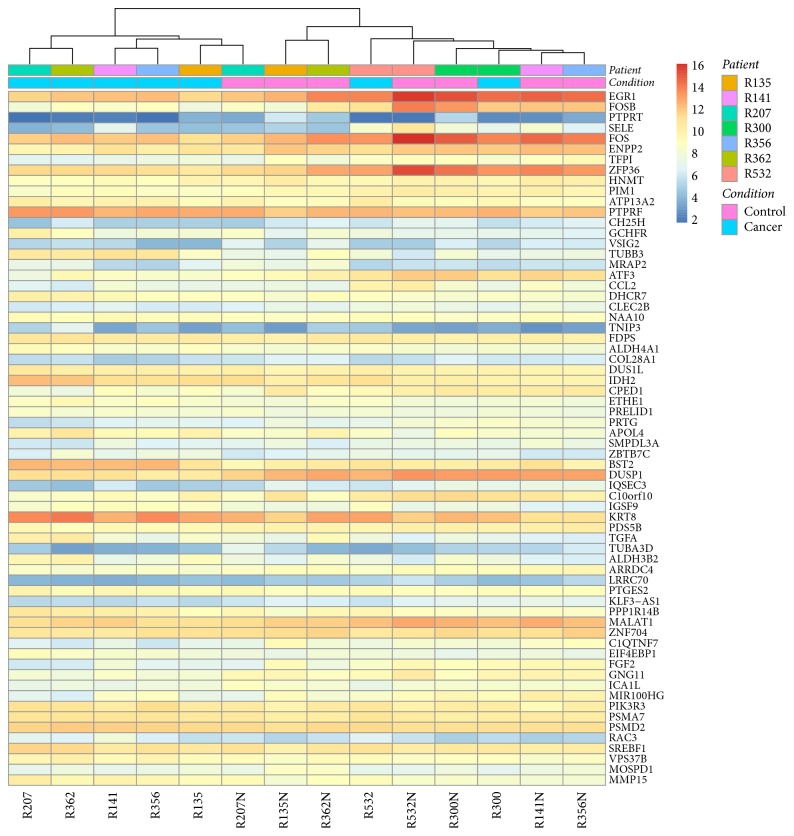
*Heat map of the top 66 differentially expressed genes*. Genes shown were selected by the adjusted *p* value < 0.01 in ascending order in the output of DESeq2 analysis on RNA-seq data from 7 pairs of samples (cancer or control). Colored legend indicates patient code, condition (cancer or control), and the relative expression level.

With the first approach, when comparing cancer tissues with noncancer controls, a total of 665 genes (357 up and 308 down) were identified as differentially expressed at the statistical significance level of adjusted (for multiple comparison) *p* value < 0.05. The entire list of differentially expressed genes is given in Suppl.  Table 2. On the top of gene list ranked by ascending adjusted *p* value there are* EGR1, FOSB, PTPRT, SELE, FOS, ENPP2, TFPI, ZFP36, *and others. Those with adjusted *p* value < 0.01 and log2 fold change (L2FC) >2 were labeled in the volcano plot in Figure 1(b). TUBB3 is the most increased (L2FC = 2.177). With a cutoff at adjusted *p* value < 0.01, we obtained a list of 66 genes (Figure 2), which was used for analysis with NIH DAVID as described below.

Using the second method, the 14 samples were treated as two independent groups, cancer versus control, with pairing information ignored, then only 4 genes were found to be differentially expressed at adjusted *p* value < 0.05, including* IGSF9, c10orf35*, and* ZNF710* genes overexpressed and the* HHATL* gene downregulated in cancer versus control. The normalized counts of these 4 genes were plotted in Figures 1(c)–1(f) and the full list of genes is given in Suppl.  Table 3. Reanalysis of the RNA-seq study by Xiong et al. on paired cancer and normal tissues from 3 endometrial cancer patients produced another list of differentially expressed genes (Suppl.  Table 4) [9].

**Figure 3:**
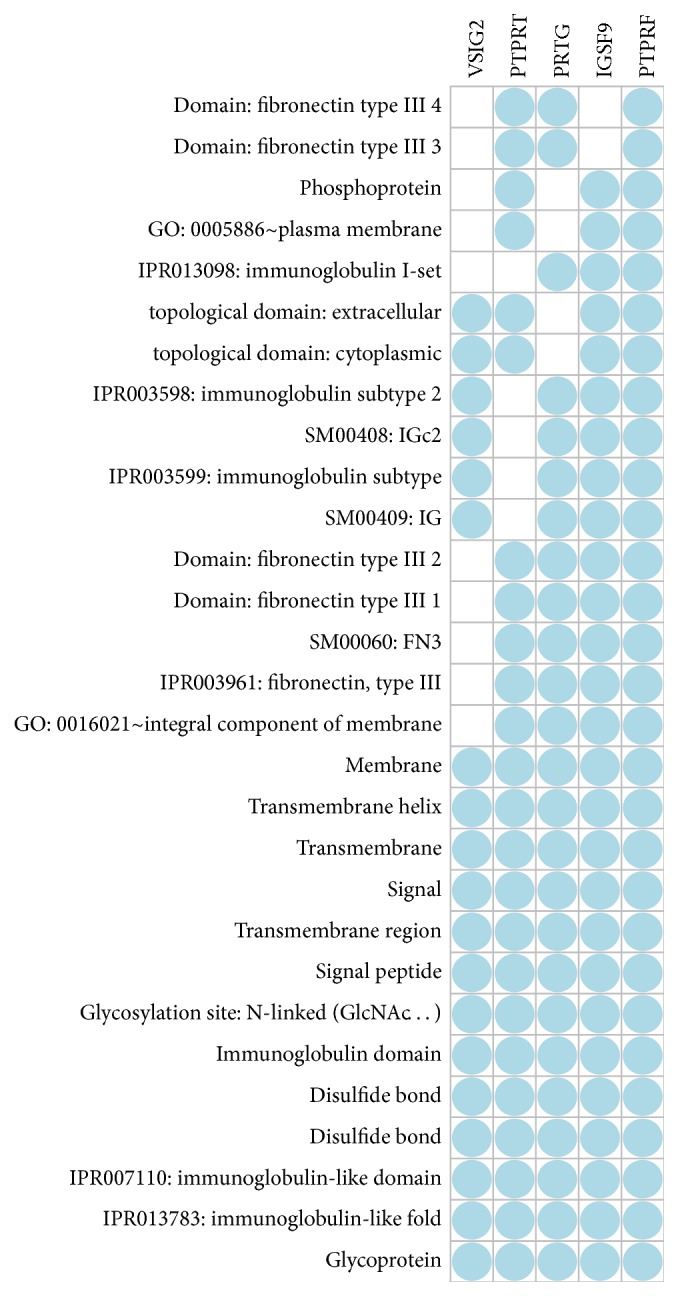
*Gene functional classification*. With either medium or high stringency, the gene functional classification tool from NIH DAVID bioinformatics resource produced only one cluster consisting of 5 genes when fed with 66 genes at adjust *p* value < 0.01 as shown in the Supplementary Table 2. Columns are genes names and rows show the related functional categories shared by the genes indicated by the blue dots.

With the aforementioned list of top 66 genes that were taken from the differentially expressed genes cutoff at adjusted *p* value < 0.01, we performed the analysis with the gene function classification tool from NIH DAVID [17], which has the capability of addressing the enriched and redundant relationships among many-genes-to-many-terms and can reduce the complexity of gene list. Out of the 66 genes, 5 genes, that is,* VSIG2, PTPRT, PRTG, IGSF9*, and* PTPRF *(Figure 3), were found to share functional similarity and form the only enriched cluster, with an enrichment sore of 0.96. Among them,* IGSF9* is the one that increased most in terms of log2 folds. This result from the gene function classification assay with DAVID plus the short list of differentially expressed genes we obtained when treating the samples as 2 independent groups prompted us to further evaluate the expression of IGSF9 with immunohistochemistry on human endometrial cancer tissues.

### 3.2. IGSF9 Expression in Endometrial Tissue and Endometrioid Carcinoma

**Figure 4:**
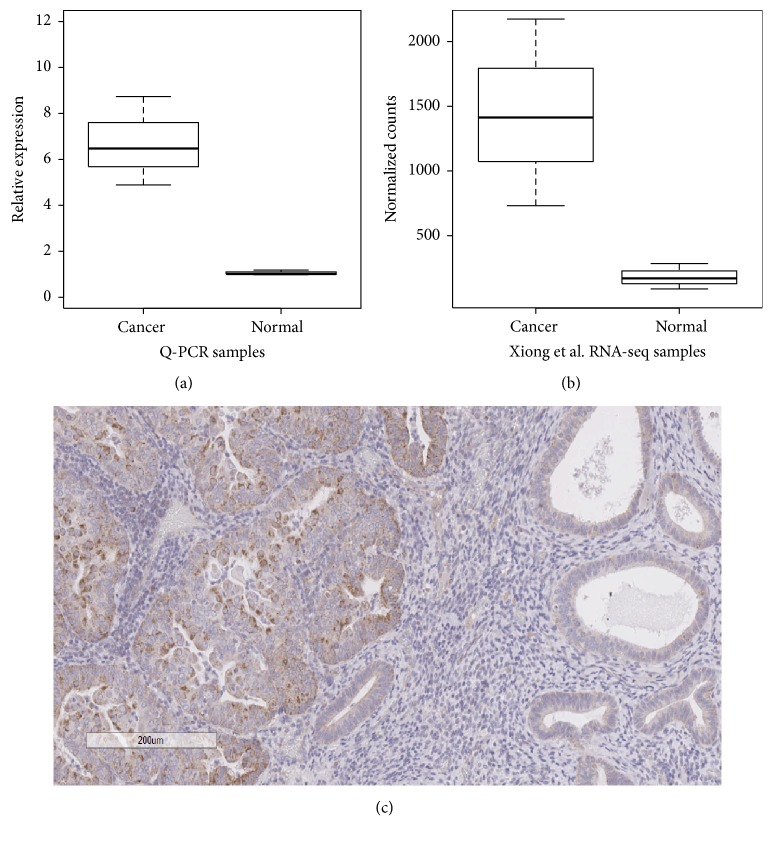
*Expression of IGSF9 in normal endometrium and endometrial carcinoma*. (a) Q-PCR measurement of* IGSF9* from RNA samples of three paired cancer and normal tissues; the Wilcoxon signed rank test gives *p* value = 0.25, while the paired *t*-test gives *p* value = 0.038. (b) Reanalysis of published RNA-seq data from Xiong et al. [9]; the Wald test gives *p* value = 6.80*E* − 10 and adjusted *p* value = 1.27*E* − 07. (c) IHC staining of IGSF9, the right half is noncancerous endometrial glands with weak brown stain, while the left side cancer cells stained strongly brown indicating higher level of expression.

The first study on IGSF9 expression was on the developing nervous system in mouse and human [18]; however to date there are no published data detailing IGSF9 expression in endometrial tissue. We first performed quantitative PCR to confirm the differential expression of IGSF9 in cancer versus noncancer endometrial tissues. With 3 paired RNA samples from the RNA-seq project, the relative expression in cancer tissue was found to be over 6 folds compared to adjacent tissue (Figure 4(a)). IGSF9 is also one of differentially expressed genes identified by our reanalysis of the RNA-seq data from Xiong et al. For side-by-side comparison, we plotted their normalized counts (Figure 4(b)).

As further characterization of IGSF9 expression in relation to cancer microscopic features requires immunohistochemistry, we evaluated IGSF9 expression at protein level in FFPE materials from 56 patients. Normal endometrial stroma and glands usually stain negative or weak while endometrioid carcinoma stained stronger (Figure 4(c)). The staining pattern is both membranous and cytoplasmic, but not the entire membrane, with uneven distribution inside the cells, often concentrated on one end of the stained cells. Some specimens show a punctate and particulate perinuclear staining pattern.

### 3.3. Correlation of IGSF9 Expression with Clinical Pathologic Features

**Figure 5:**
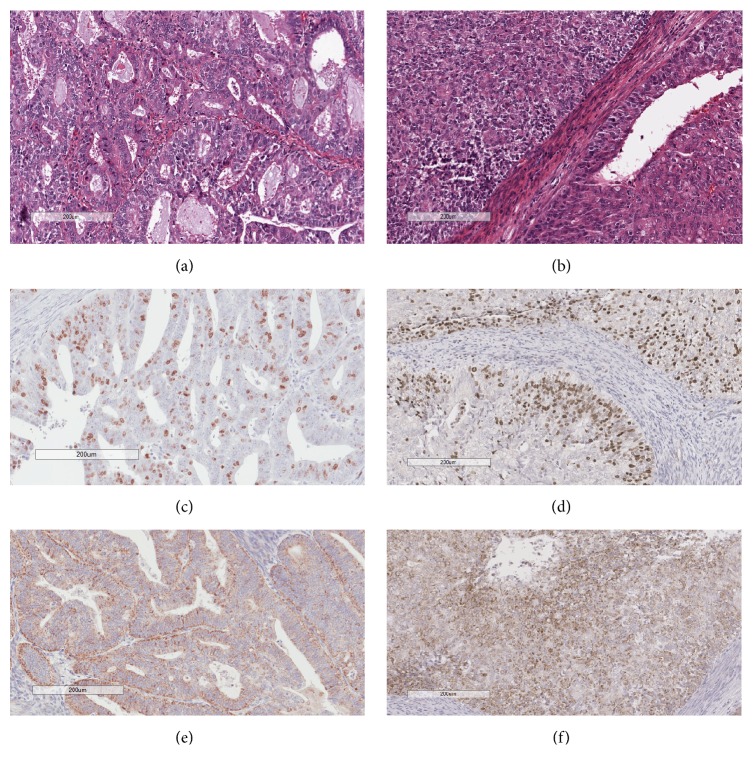
*Histopathological and immunohistochemical evaluation of endometrial carcinoma*. (a) and (b) H&E stain of endometrioid endometrial carcinoma, histological grades I and II, respectively; (c) and (d) Ki67 stain on grade I and II sections, respectively; (e) and (f) IGSF9 stain on grade I and II sections, respectively. Both immunostains were quantified with the image analysis tools from Aperio digital pathology system. Numeric data were used in statistical analysis.

The association of IGSF9 expression with clinicopathologic characteristics of these patients was systematically examined in our collection of 56 patients with endometrial cancer, which were aged from 39 to 94 years, with a median of 64. All patients were diagnosed as endometrioid carcinoma, 28 at grade I, and 28 at grade II in histologic grading (Table 1, Figures 5(a)-5(b)) [19]. As for clinical stages, 46 patients were at stage I, three at stage II, and seven at stage III. Tumor size, the depth of myometrial invasion, tumor cell labeling of proliferation marker Ki67 (Figures 5(c)-5(d)), and the presence or absence of lymphovascular invasion were recorded as part of clinical and pathologic data.

**Figure 6:**
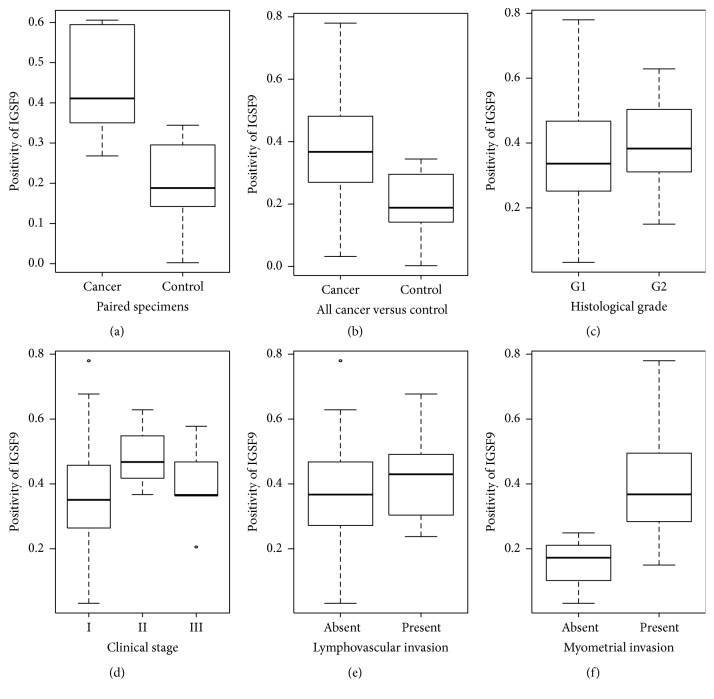
*IGSF9 expression in association with categorical clinicopathologic features*. (a) Comparison of IGSF9 positivity in 8 pairs endometrioid carcinoma and noncancerous endometrial epithelia (*p* value = 0.008). (b) Comparison between noncancerous tissues and all the endometrioid carcinoma tissues from 56 patients (*p* value = 0.002). (c) Histological grade I (G1) versus grade II (G2). (d) Clinical stages. (e) Lymphovascular invasion. (f) Myometrial invasion (*p* value = 0.013).

The positivity of IGSF9 expression as determined by immunohistochemistry (Figures 5(e)-5(f)) was presented in decimal format. Among the cases evaluated were eight patients for which both cancer and control (normal/noncancerous) tissues were available. Paired comparison of relative IGSF9 expression shows significantly enhanced staining in cancer tissues relative to controls with values of 0.448 versus 0.200, respectively (*p* value = 0.008 by the Wilcoxon test) (Figure 6(a)). Comparison of the all the cancer tissues with the 8 control tissues showed a similar trend (Figure 6(b)) with statistical significance (*p* value = 0.002 by the Wilcoxon test).

A trend was observed in which IGSF9 positivity is higher in histological grade II (G2) relative to grade I (G1) (Figure 6(c)), but the results are not statistically significant. No association was found in relation to clinical staging and lymphovascular invasion (Figures 6(d) and 6(e)). Interestingly, however, statistically significant difference was obtained when comparing the IGSF9 positivity between patients without any degree of myometrial invasion and those with myometrial invasion (0.151 versus 0.395; *p* value = 0.013 by the Wilcoxon test) (Figure 6(f)).

**Figure 7:**
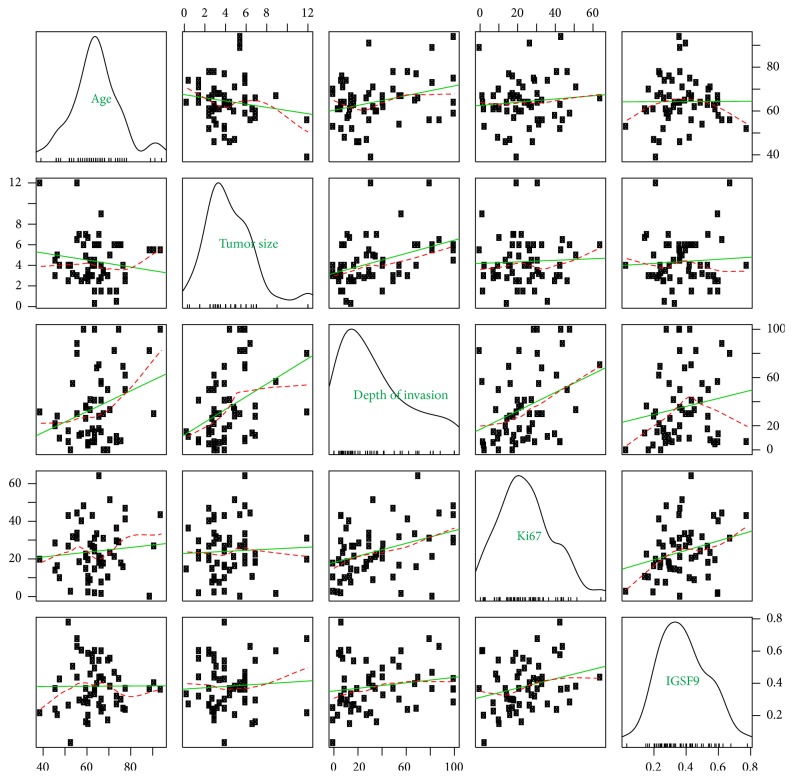
*IGSF9 expression in association with continuous clinicopathologic features*. Scatter plots showing the pairwise correlations of age (years), tumor size (diameter in cm), depth of myometrium invasion (percentage of the entire myometrium thickness), Ki67 label index (positive percentage of tumor cells), and IGSF9 positivity (positive percentage of analyzed pixels) with each other in the off-diagonals. Loess smoothed (red) and linear (green) fit lines were superimposed on these plots. Each parameter was named in the principal diagonal overlaid with density and rug plots of that parameter. Depth of myometrial invasion correlated with tumor size (coefficient = 0.412, *p* value = 0.020) and Ki67 labeling index (coefficient = 0.360, *p* value = 0.006).

The correlation of IGSF9 positivity with other continuous variables, such as age, tumor size in cm, depth of myometrium invasion as percentage of total myometrium thickness, and Ki67 labeling index were plotted in Figure 7. IGSF9 expression has somewhat of a correlation with Ki67 labeling index, but not statistically significant. However the positive correlation of tumor size with depth of myometrium invasion (coefficient = 0.412, *p* value = 0.002) and that of Ki67 with depth of myometrium invasion (coefficient = 0.360, *p* value = 0.006) were statistically significant.

### 3.4. IGSF9 Expression Status in RNA-seq Data from TCGA

As a way of in silico validation, we surveyed the expression of* IGSF9* in TCGA RNA-seq dataset via the web portal of UALCAN [14]. As of November 2017, normalized RNA expression data from 33 types of cancer are available, but not all have proper amount of normal controls for comparison.* IGSF9 *had higher expression in uterine corpus endometrial cancer (UCEC) tissues and 7 other types of cancers compared to their corresponding normal controls (Table 2). In contrast,* IGSF9* expression is lower in cancer versus normal control in rectum adenocarcinoma and colon adenocarcinoma (Table 2).

**Figure 8:**
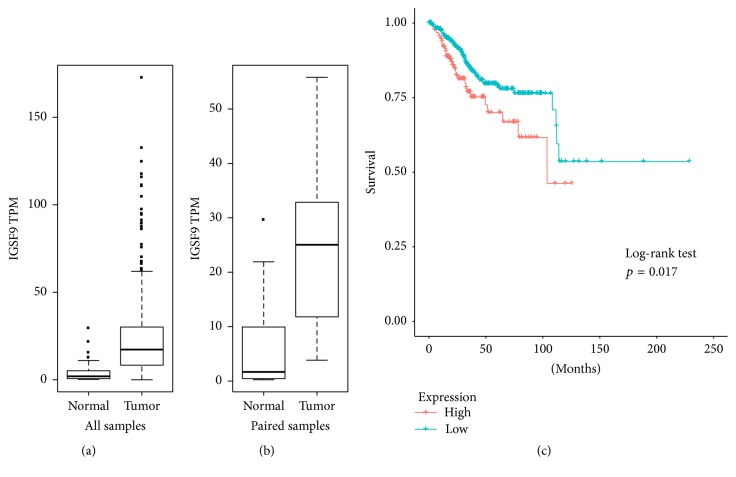
*IGSF9 expression in TCGA endometrial cancer project*. (a) Normalized RNA-seq data for* IGSF9*, transcripts per million (TMP), clinical data of 546 patients cancer specimen versus 35 normal tissues (*p* value < 0.001). (b) comparison of 23 patients with RNA-seq data from both cancer tissue and normal control (*p* value < 0.001). (c) Kaplan-Meier survival analysis of 543 patients with IGSF9 expression and outcome data. Log-rank test, *p* value = 0.017.

UALCAN contains reprocessed RNA-seq data from 546 UCEC tissues and 35 normal endometrial tissues. Out of that, 23 patients provided both cancer and normal tissues.* IGSF9* mRNA expression was found to be higher in both the comparison of all cancer versus normal control (*p* < 0.001 by Wilcoxon test) and comparison of paired cancer versus normal from the same patients (*p* < 0.001 by Wilcoxon test) (Figures 8(a) and 8(b)).

A total of 543 endometrial cancer patients with RNA-seq and overall outcome data were used for Kaplan-Meier survival analysis. Overexpression of* IGSF9* (top 25%) was associated with poor survival (Figure 8(c), log-rank test, *p* = 0.017). A statistically significant association of* IGSF9* overexpression and poor overall survival was also observed in thymoma, skin cutaneous melanoma, and brain lower grade glioma (Table 2).

## 4. Discussion

To find differentially expressed genes between groups of specimens, RNA-seq is a very powerful tool as it is high throughput and reliable in quantification [10, 20]. For gene expression profiling with RNA-seq, TCGA has data from the largest number of patients [2]. The reports and datasets from TCGA are a rich resource for evaluation of genetic variations or molecular markers of endometrial cancer. Based on their comprehensive findings on somatic copy number alterations and exome sequencing, a new molecular classification system was proposed for endometrial cancer [7]. However, limited evaluation of the mRNA expression data was provided [7, 8]. By design, the raw RNA-seq data and clinicopathologic characteristics in all the TCGA projects are open to researchers who are interested in further analyses. Noticeably, many secondary online resources based on TCGA datasets (e.g., the UALCAN website) have recently appeared and greatly eased the use of these data [14, 21]. The report by Dellinger and colleagues utilized TCGA data to show that L1CAM is an independent predictor of poor survival in endometrial cancer [22].

The lack of information from normal tissues or benign lesions in RNA-seq data from the initial TCGA endometrial cancer publication piqued our interest and others as well. Xiong and colleagues used RNA-seq to evaluate both mRNA and microRNA expression profiles in three patients with both cancer and noncancer tissues [9]. In addition to mRNA and microRNA expression profiles, their study also performed genetic variation calling from RNA-seq data, which is a feasible but less established approach [23].

In the current study, our RNA-seq with paired cancer and control tissues from 7 endometrioid carcinoma patients discovered a unique set of differentially expressed genes. For example, our reanalysis of Xiong's data identified* WISP2 (CCN5)* (log2 fold change −7.12; Suppl.  Table 4) as a highly differentially expressed gene. It is reported to be a tumor suppressor; the deficiency of* WISP2 *promotes breast cancer growth [24] and* WISP2* RNA expression was decreased in 79% of human colon cancers [25], but no studies of* WISP2* in endometrial cancer have been reported. The* TUBB3* gene is the most increased gene on our list of genes (Figure 1(b)) from our own patient materials.* TUBB3* encodes class III *β*-tubulin and its overexpression has been linked to resistance to paclitaxel and correlated with poor survival in ovarian, breast, gastric, and non-small-cell lung cancers and unknown primary tumors [26, 27]. This finding was not confirmed in endometrial cancer and class III *β*-tubulin expression was also not correlated with clinicopathologic characteristics [27, 28].

Facing the long list of differentially expressed genes, tools for functional profiling are often used to extract biological meaning from such data. RNA-seq specific analysis packages like GOseq and SeqGSEA are available [29]. Our use of the gene functional classification tool in the NIH DAVID package and our top 66 genes provided only one cluster of genes, and treating our paired samples as two independent groups further narrowed the gene list. As* IGSF9* is the only common gene from both analyses, we focused on IGSF9 and explored the association of IGSF9 protein expression with clinicopathological characteristics in endometrioid endometrial carcinoma.

IGSF9 was first cloned and characterized as a member of the immunoglobulin superfamily expressed in a wide variety of tissues in human and mouse [18]. Structurally, it contains five Ig domains, two fibronectin III domains, a single transmembrane domain, and an intracellular C-terminal and it was found to be related to dendrite arborization in the brain [30]. To date, there are no published reports on IGSF9 in any type of cancer tissue. Existing studies of IGSF9 are very limited (10 papers in PubMed as of January 2018). Our study is the first to investigate the association of its expression with cancer, but its expression and mutation profiles could be assessed via searching online databases, such as the Human Protein Atlas project (http://www.proteinatlas.org), GTEx (https://GTExPortal.org), and the Catalog of Somatic Mutations in Cancer (COSMIC, http://cancer.sanger.ac.uk/cosmic).

The expression pattern as revealed by our immunohistochemical analyses in endometrioid carcinoma confirms the localization of IGSF9 on the cellular membrane and in the cytoplasm. We confirmed the differential expression of IGSF9 in cancer versus noncancer tissues with materials from a cohort of 56 patients. The expression of IGSF9 is higher in cancer than that in normal or benign endometrial tissue. The positivity of IGSF9 is also higher in cancer with myometrium invasion than those without, likely an indicator of cancer aggressiveness. However, as these observations were based on the small and retrospective cohort of patients available, further studies on larger and prospective cohorts were needed. Analysis of the expression data from TCGA as we surveyed with UALCAN provided the necessary support. These data confirmed that* IGSF9 *is overexpressed in cancerous versus normal endometrial tissue as well as in 7 additional major cancer types as well. Interestingly we observed the opposite relationship in colorectal cancer, wherein* IGSF9* was expressed at lower levels in cancer versus normal, suggesting a topic to be further studied.

A limitation of the current study is our lack of access to survival data for our local cohort of 56 patients. However, analysis of publicly available TCGA RNA-seq data via UALCAN provided further support for our initial finding and enabled survival analyses. Overexpression of* IGSF9* is an indicator of poor prognosis in endometrial cancer. Moreover,* IGSF9* overexpression is also associated with poor prognosis in several other cancer types, including brain lower grade glioma, skin cutaneous melanoma, and thymoma, suggesting that the significance of* IGSF9* in cancer pathology is likely not limited to endometrial cancer.

## 5. Conclusions

We identified via RNA-seq a list of differentially expressed genes in endometrioid endometrial carcinomas versus noncancer controls. Focusing on* IGSF9*, an adhesion molecule that has not been characterized in connection with cancer pathology, we confirmed the overexpression of IGSF9 in endometrial cancer and found an association with myometrial invasion and poor outcome. These studies provide preliminary support for consideration of IGSF9 as a new biomarker in endometrial cancer with potential utility in diagnostics, prognostics, and therapeutics. Further studies on the role of IGSF9 in cancer pathology are warranted.

## Figures and Tables

**Table 1:** Clinicopathological characteristics of patients in IHC study.

Parameters	Category/measurement	
Age, mean ± SD, range (year)	64.3 ± 10.7 (39–94)	
Tumor size, mean ± SD, range (cm)	4.34 ± 2.33 (0.3–12)	
Clinical stage	I	46
	II	3
	III	7
Histologic type	Endometrioid	56
	Serous	0
Histologic grade	G1	28
	G2	28
	G3	0
Myometrium invasion	Absent	3
	Present	53
Lymphovascular invasion	Absent	49
	Present	7

**Table 2:** IGSF9 expression and survival correlation in TCGA RNA-seq dataset.

Abbrev.	Cancer type	Sample size^(1)^	Expression level^(2)^	Overall survival^(3)^
BLCA	Bladder urothelial carcinoma	Normal (*n* = 19) Tumor (*n* = 408)	Up	-
BRCA	Breast invasive carcinoma	Normal (*n* = 114) Tumor (*n* = 1094)	Up	-
CESC	Cervical squamous cell carcinoma and endocervical adenocarcinoma	Normal (*n* = 3) Tumor (*n* = 305)	Up	-
CHOL	Cholangiocarcinoma	Normal (*n* = 9) Tumor (*n* = 36)	Up	-
COAD	Colon adenocarcinoma	Normal (*n* = 41) Tumor (*n* = 286)	Down	-
HNSC	Head and neck squamous cell carcinoma	Normal (*n* = 44) Tumor (*n* = 520)	Up	-
LGG	Brain lower grade glioma	Grade II (*n* = 248) Grade III (*n* = 265)	-	Yes
LUSC	Lung squamous cell carcinoma	Normal (*n* = 59) Tumor (*n* = 515)	Up	-
READ	Rectum adenocarcinoma	Normal (*n* = 11) Tumor (*n* = 166)	Down	-
SKCM	Skin cutaneous melanoma	Normal (*n* = 1) Tumor (*n* = 472)	-	Yes
THCA	Thyroid carcinoma	Normal (*n* = 60) Tumor (*n* = 505)	Up	-
THYM	Thymoma	Normal (*n* = 2) Tumor (*n* = 121)	-	Yes
UCEC	Uterine corpus endometrial cancer	Normal (*n* = 35) Tumor (*n* = 546)	Up	Yes

*Notes*. ^(1)^The number of normal versus tumor samples, except for LGG. ^(2)^“Up” or “Down” indicates significant expression level changes, tumor versus normal; dash indicates no significant change. ^(3)^Kaplan-Meier analysis; “Yes” indicates that higher expression of IGSF9 is related to poor overall survival.

## Abbreviations

IGSF9: Immunoglobulin Super Family 9
NGS: Next-generation sequencing
RNA-seq: RNA-sequencing
SEER: Surveillance, Epidemiology, and End Results
ICGC: International Cancer Genomics Consortium
TCGA: The Cancer Genome Atlas
FFPE: Formalin-fixed and paraffin-embedded
IHC: Immunohistochemistry
Q-PCR: Quantitative Polymerase Chain Reaction
COSMIC: Catalog of Somatic Mutations in Cancer.

## Supplementary Materials

Supplementary 1 Supplementary Table 1: QC and alignment results.

Supplementary 2 Supplementary Table 2: differentially expressed genes as paired tissues.

Supplementary 3 Supplementary Table 3: differentially expressed genes as independent groups.

Supplementary 4 Supplementary Table 4: differentially expressed genes from reanalyzing data of Xiong et al.

## References

[1] Marioni J. C., Mason C. E., Mane S. M., Stephens M., Gilad Y. (2008). RNA-seq: an assessment of technical reproducibility and comparison with gene expression arrays. *Genome Research*.

[2] Tomczak K., Czerwińska P., Wiznerowicz M. (2015). The Cancer Genome Atlas (TCGA): An immeasurable source of knowledge. *Wspolczesna Onkologia*.

[3] Chin L., Hahn W. C., Getz G., Meyerson M. (2011). Making sense of cancer genomic data. *Genes & Development*.

[4] International Cancer C., Genome T. J., Anderson W. International network of cancer genome projects, Nature.

[5] Le Gallo M., O'Hara A. J., Rudd M. L. (2012). Exome sequencing of serous endometrial tumors identifies recurrent somatic mutations in chromatin-remodeling and ubiquitin ligase complex genes. *Nature Genetics*.

[6] Torre L. A., Bray F., Siegel R. L., Ferlay J., Lortet-Tieulent J. (2015). Global cancer statistics, 2012. *CA: A Cancer Journal for Clinicians*.

[7] Kandoth C., Schultz N., Cherniack A. D. (2013). Integrated genomic characterization of endometrial carcinoma. *Nature*.

[8] Sun H., Yan L., Tu R. (2017). Expression Profiles of Endometrial Carcinoma by Integrative Analysis of TCGA Data. *Gynecologic and Obstetric Investigation*.

[9] Xiong H., Li Q., Liu S. (2014). Integrated microRNA and mRNA transcriptome sequencing reveals the potential roles of miRNAs in stage I endometrioid endometrial carcinoma. *PLoS ONE*.

[10] Anders S., Huber W. (2010). Differential expression analysis for sequence count data. *Genome Biology*.

[11] Anders S., McCarthy D. J., Chen Y. (2013). Count-based differential expression analysis of RNA sequencing data using R and Bioconductor. *Nature Protocols*.

[12] Yu G., He Q.-Y. (2016). ReactomePA: An R/Bioconductor package for reactome pathway analysis and visualization. *Molecular BioSystems*.

[13] Huang D. W., Sherman B. T., Lempicki R. A. (2009). Systematic and integrative analysis of large gene lists using DAVID bioinformatics resources. *Nature Protocols*.

[14] Chandrashekar D. S., Bashel B., Balasubramanya S. A. H. (2017). UALCAN: A Portal for Facilitating Tumor Subgroup Gene Expression and Survival Analyses. *Neoplasia (United States)*.

[15] Shi Z., Liu Y., Johnson J. J., Stack M. S. (2011). Urinary-type plasminogen activator receptor (uPAR) modulates oral cancer cell behavior with alteration in p130cas. *Molecular and Cellular Biochemistry*.

[16] Shi Z., Stack M. (2015). An Update on Immunohistochemistry in Translational Cancer Research. *Cancer Translational Medicine*.

[17] Huang D. W., Sherman B. T., Tan Q. (2007). The DAVID gene functional classification tool: a novel biological module-centric algorithm to functionally analyze large gene lists. *Genome Biology*.

[18] Doudney K., Murdoch J. N., Braybrook C. (2002). Cloning and characterization of Igsf9 in mouse and human: A new member of the immunoglobulin superfamily expressed in the developing nervous system. *Genomics*.

[19] Reichert R. A. (2012). *Diagnostic gynecologic and obstetric pathology: an atlas and text*.

[20] Conesa A., Madrigal P., Tarazona S. (2016). A survey of best practices for RNA-seq data analysis. *Genome Biology*.

[21] Zhu Y., Qiu P., Ji Y. (2014). TCGA-assembler: Open-source software for retrieving and processing TCGA data. *Nature Methods*.

[22] Dellinger T. H., Smith D. D., Ouyang C., Warden C. D., Williams J. C., Han E. S. (2016). L1CAM is an independent predictor of poor survival in endometrial cancer - An analysis of The Cancer Genome Atlas (TCGA). *Gynecologic Oncology*.

[23] Sun Z., Bhagwate A., Prodduturi N., Yang P., Kocher J. A. Indel detection from RNA-seq data: tool evaluation and strategies for accurate detection of actionable mutations. *Briefings in Bioinformatics*.

[24] Das A., Dhar K., Maity G. (2017). Deficiency of CCN5/WISP-2-Driven Program in breast cancer Promotes Cancer Epithelial cells to mesenchymal stem cells and Breast Cancer growth. *Scientific Reports*.

[25] Pennica D., Swanson T. A., Welsh J. W. (1998). WISP genes are members of the connective tissue growth factor family that are up-regulated in wnt-1-transformed cells and aberrantly expressed in human colon tumors. *Proceedings of the National Acadamy of Sciences of the United States of America*.

[26] English D. P., Roque D. M., Santin A. D. (2013). Class III b-tubulin overexpression in gynecologic tumors: Implications for the choice of microtubule targeted agents?. *Expert Review of Anticancer Therapy*.

[27] Vandenput I., Capoen A., Coenegrachts L. (2011). Expression of ERCC1, p53, and Class III *β*-tubulin do not reveal chemoresistance in endometrial cancer results from an immunohistochemical study. *International Journal of Gynecological Cancer*.

[28] Zhu C., Luo J., Shi H. (2009). Expression of tubulin, p53, ki67, receptors for estrogen, and progesterone in endometrial cancer. *Eur J Gynaecol Oncol*.

[29] Young M. D., Wakefield M. J., Smyth G. K., Oshlack A. (2010). Gene ontology analysis for RNA-seq: accounting for selection bias. *Genome Biology*.

[30] Shi S.-H., Cox D. M., Wang D., Jan L. Y., Jan Y.-N. (2004). Control of dendrite arborization by an Ig family member, dendrite arborization and synapse maturation 1 (Dasm1). *Proceedings of the National Acadamy of Sciences of the United States of America*.

